# Response of One-Carbon Biomarkers in Maternal and Cord Blood to Folic Acid Dose During Pregnancy

**DOI:** 10.3390/nu16213703

**Published:** 2024-10-30

**Authors:** Jennifer M. Fleming, Gisselle Rosa, Victoria Bland, Gail P. A. Kauwell, Olga V. Malysheva, Alleigh Wettstein, Dorothy B. Hausman, Lynn B. Bailey, Hea Jin Park

**Affiliations:** 1 Department of Nutritional Sciences, University of Georgia, Athens, GA 30602, USA; 2 Department of Health Sciences, University of Central Florida, Orlando, FL 32816, USA; 3 Division of Nutritional Sciences, Cornell University, Ithaca, NY 14850, USA

**Keywords:** unmetabolized folic acid, folate, supplementation

## Abstract

Background/Objectives: The folate Recommended Daily Allowance (RDA) for pregnant women is 600 μg/day dietary folate equivalents, which is equivalent to approximately 400 μg folic acid. Many prenatal supplements contain much higher doses of folic acid. The body’s ability to reduce synthetic folic acid to the metabolically active form may be exceeded with high levels of supplementation. The objective of this double-blinded randomized controlled intervention trial was to determine changes in unmetabolized folic acid and other biomarkers of folate and one-carbon metabolism in maternal and cord blood in response to a folic acid dose commonly found in prenatal supplements (800 μg/day) compared to the dose equivalent of the RDA (400 μg/day). Methods: Healthy pregnant women were randomized and provided supplements from their first prenatal visit (<12 weeks gestation) through delivery. Maternal blood was collected at baseline and delivery. Umbilical cord blood from the mothers was collected at delivery. Results: A repeated measures analysis of variance revealed that there was a significant group supplemental dose effect (*p* = 0.0225) on serum unmetabolized folic acid concentration in mothers but no difference in cord blood unmetabolized folic acid concentrations between groups. Mixed effects analysis found a significant overall effect of pre-pregnancy BMI (*p* = 0.0360) and length of previous folic acid supplementation (*p* = 0.0281) on serum folate concentrations. No treatment effect was seen in RBC folate concentrations. Choline concentrations were higher in cord blood from the 800 μg/day group compared to the 400 μg/day group, but there was no group effect in maternal choline concentrations. Conclusions: The results indicate that folic acid dose during pregnancy affects certain folate and one-carbon biomarkers, and these effects are not consistent between maternal and cord blood. Potential long-term effects of these results on both mothers and offspring are unknown and merit further investigation.

## 1. Introduction

Folate is a water-soluble B vitamin that exists naturally in certain foods. The synthetic form is folic acid, which is found in fortified foods and dietary supplements. Naturally occurring folate is in the reduced form while folic acid is the fully oxidized form [1]. Folate metabolites function as coenzymes that transfer one-carbon units by accepting and donating them [2]. Because of folate’s key role in one-carbon metabolism, which is essential for DNA and protein synthesis, the body’s requirement for folate increases when there is an increase in cellular division, such as during pregnancy [3]. Failing to consume adequate amounts of folate from one’s diet and supplements can have serious negative consequences, including developmental disorders such as neural tube defects (NTDs) [4]. Thus, folic acid supplementation is encouraged for women of childbearing age and pregnant women to ensure that they are consuming adequate folate.

The Recommended Dietary Allowance for pregnant women is 600 μg dietary folate equivalents (DFEs) per day. One μg DFE is equal to 1 μg of food folate or approximately 0.6 μg of folic acid due to the greater bioavailability of folic acid compared to food folate [5]. Although the recommendation for pregnant women is to consume 400 μg of folic acid per day and emphasizes periconceptual folic acid supplementation, the folic acid content of most over-the-counter prenatal supplements is at least twice that amount [6].

Folic acid must be converted to its reduced form to function as a coenzyme in metabolic processes [7]. At high levels of intake, the body’s capacity to reduce folic acid may be exceeded, leading to detectable circulating levels of unmetabolized folic acid [8]. Unmetabolized folic acid was noted to be present in most serum samples from children, adolescents, and adults participating in the 2007–2008 NHANES cross-sectional study [9]. Additionally, unmetabolized folic acid has been detected in cord blood samples, although research in this area is limited [10,11].

During pregnancy, folic acid is transferred to the fetus via the placenta [12]. Studies in both humans and animals indicate the possibility of a protective mechanism that prevents the fetus from receiving toxic amounts of certain vitamins, even in the presence of high maternal intakes and serum concentrations [13,14]. There is rising concern, however, that there are potential harmful consequences associated with excess prenatal folic acid intake [15]. A recent review by Xu et al. addresses such concerns, highlighting adverse effects on offspring of mothers who present with high folic acid levels [16]. Notably, Raghavan et al. assessed autism spectrum disorder (ASD) as a health consequence associated with excessive unmetabolized folic acid cord blood concentrations [17]. The observational Boston Birth Cohort investigation expanded on the authors’ previous work that showed a U-shaped curve of ASD risk in offspring whose mothers supplemented at low (<2 times/week), moderate (3–5 times/week), and high (>5 times per week) frequencies; moderate intake was associated with decreased incidence of ASD; low and high intakes were associated with increased risk [17]. A potential mechanism is the influence of folic acid fortification on methylation patterns and gene expression in the developing brain that parallels changes seen in ASD [16,17]. The findings suggest that increased levels of folic acid may pose significant risks to children whose brains are compromised during development, as in cases of prematurity and sex-related neurological disability susceptibility [18].

This study aims to investigate whether there are differences in circulating concentrations of unmetabolized folic acid in pregnant women consuming the recommended dose of folic acid (400 μ/day) compared to the dose commonly found in over-the-counter prenatal supplements (800 μ/day) and whether these differences affect the infant. Based on previous research findings, it was hypothesized that maternal concentrations of unmetabolized folic acid would be higher in the group exposed to the higher dose of folic acid [9,10], while cord blood concentrations would not differ between groups [10,19].

## 2. Materials and Methods

### 2.1. Participant Recruitment and Inclusion/Exclusion Criteria

**Figure 1:**
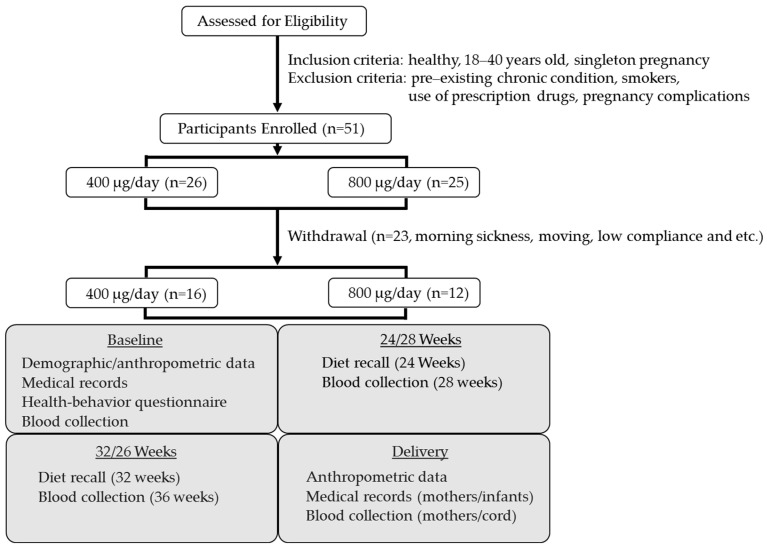
Flow diagram for study participants.

A flow diagram for study participants is shown in Figure 1. Participants were recruited by midwives from Athens Regional Midwifery Clinic (ARMC) at their initial prenatal visit (<12 weeks gestation). The midwife recruiters attended several in-service training sessions covering folate, its role in pregnancy, the study purpose and design, participant inclusion and exclusion criteria, and their role in the study. They also completed the Collaborative Institutional Training Initiative (CITI) on human subjects. Inclusion criteria for study participants included healthy 18 to 40-year-old pregnant women with singleton pregnancies who were willing to comply with study protocols. Exclusion criteria were a pre-existing chronic condition including anemia, diabetes and hypertension; smokers; use of prescription drugs; complications associated with pregnancy such as pre-eclampsia and gestational diabetes; and carrying more than one fetus. Participants were not allowed to take any prenatal supplements other than those provided by the research team for the study after study enrollment. The midwives obtained written informed consent from all eligible and willing women before they were enrolled in the study. All methods and procedures were approved by the University of Georgia Institutional Review Board on Human Subjects (STUDY00000506) and the Athens Regional Medical Center Institutional Review Board before the study began. The study was registered at ClinicalTrials.gov (NCT02124642).

### 2.2. Folic Acid Supplement Protocol

This study was performed as a double-blinded randomized controlled intervention trial. Participants were randomized into two treatment groups at enrollment, one group supplemented with 400 μg of folic acid and the other 800 μg of folic acid daily throughout pregnancy until delivery. All participants were provided with three tablets/capsules daily including a multivitamin/multimineral tablet (One-A-Day^®^ Women’s, Bayer’s Healthcare, Somerville, NJ, USA) that contained 400 μg of folic acid along with other vitamins and minerals required for pregnant women, a capsule containing 200 mg docosahexaenoic acid (DHA) (Life’s DHA™ 200 mg vegetarian capsules, DSM Nutritional Products North America, Parsippany, NJ, USA), and a specially formulated capsule containing 10 mg of iron and either 0 or 400 μg of folic acid, resulting in a total daily intake of either 400 or 800 μg of folic acid per day. The specially formulated treatment capsules were compounded by Westlab Pharmacy (Gainesville, FL, USA) and were tested by third-party laboratories for folic acid (Analytical Research Laboratories, Oklahoma City, OK, USA) and iron (Covance, Madison, WI, USA) content. This supplement regimen provided the participants with vitamin and mineral amounts that are recommended for a healthy pregnancy by the Institute of Medicine. Participants were instructed to take all three supplements daily at around the same time each day. Supplements were provided at study enrollment and exchanged monthly at each follow-up prenatal visit throughout pregnancy.

### 2.3. Collection of Demographic and Anthropometric Data

Maternal demographic and anthropometric data including age, race, weight and height at baseline and throughout pregnancy were acquired from medical records. Pre-pregnancy weight was per self-report and BMI was calculated based on the weight and height. Medical records were also used to collect health information including previous pregnancies, medical history, and prescription and non-prescription drug use. Use of participant health information was authorized by signature of consent and protected by the Health Insurance Portability and Accountability Act (HIPAA). Participants completed a health-behavior questionnaire over the phone after enrollment that included information on pre-pregnancy supplementation, consumption of highly fortified foods, smoking and alcohol history, physical activity before and during pregnancy, and amount of time spent outdoors. Researchers also maintained contact with participants throughout the study via e-mail, telephone, and text message to provide information and answer questions as needed.

Infant information was obtained through medical records, including date and mode of delivery, gestational age, gender, anthropometric measurements, and Apgar scores; a subjective test used to assess clinical infant status at one and five minutes after birth.

### 2.4. Collection of Maternal Dietary Intake Data and Compliance

Participants completed their diet recall at 24 and 32 weeks gestation. Each set of recalls included three non-consecutive days of 24 h recalls: two weekdays and one weekend day. The recalls followed the format of the Automated Self-Administered 24 h dietary recall (ASA24) system. Participants were given the option to complete the paper version and return it in a pre-addressed, stamped envelope or to log in to the ASA24 system to complete the recall online. For paper versions, a trained researcher called participants once the recall was received and used questions and probes from the ASA24 program to ensure thorough recall information was obtained before entering the recall data into the ASA24 system. Nutrient analysis data using the ASA24 system are based on the Food and Nutrient Database for Dietary Surveys as described by Subar et al. [20].

Participants were contacted every two weeks via e-mail, text message, or phone call to obtain health behavior information, and to address questions about completion of dietary recalls or other questions asked by participants. Additionally, participants were reminded to return their pillboxes at their next prenatal visit and pick up their next box of supplements, which encouraged compliance to the study protocol. Compliance was measured by counting the pills returned.

### 2.5. Blood Collection and Biochemical Analysis

Non-fasting venous blood was collected at the midwifery clinic by trained ARMC staff at the participant’s initial visit, 28 weeks gestation, and 36 weeks gestation. Both maternal and cord blood were obtained at delivery at ARMC by nurses at the Labor and Delivery Unit. Cord blood was collected via the umbilical vein. Blood was collected in EDTA-coated tubes and serum separator tubes that were wrapped in foil, stored on ice, and processed within two hours of collection. Serum was allowed to clot for 30 min at room temperature before centrifuging for 15 min at 1200× *g*, after which 1 mL of serum was removed, combined with 71.4 μL of 7% ascorbic acid solution, and divided into two 500 μL aliquots for serum folate analysis. Sample preparation for analysis of serum folic acid involved the addition of 10 mg of ascorbic acid to 1 mL of serum that was then divided into two 500 μL aliquots. A 100 μL sample of whole blood was added to 1.0 mL of 1% ascorbic acid, wrapped in foil to protect from light, mixed on a rotating platform for 30 min, then divided into two 500 μL aliquots for RBC folate analysis. Complete blood count (CBC) samples were placed in biohazard sample bags and picked up by LabCorp. This analysis was performed immediately. Samples for RBC folate, serum folate, folic acid, and other one-carbon metabolite samples for analysis were stored at −80 °C prior to shipping on dry ice to analytical labs.

The *MTHFR* 677 (rs1801133) genotype was sequenced using real-time PCR by the Georgia Genomics Facility in Athens, GA, as previously described [21]. Serum and red blood cell folate concentrations were determined by microbiological assay using Lactobacillus rhamnosus [3,22]. The inter- and intra-assay coefficients of variation were 7.7% and 6.7% for serum folate, respectively, and 5.1% and 3.3% for RBC folate, respectively. Oxidized serum folic acid was analyzed using LC-MS/MS stable isotope-dilution methods as described by West et al. [23]. The LC-MS/MS system was a TSQ Quantum mass spectrometer (Thermo Fisher Scientific, Waltham, MA, USA) with refrigerated Accela autosampler (Thermo Fisher Scientific, Waltham, MA, USA) and Accela pump with degasser (Thermo Fisher Scientific, Waltham, MA, USA). Plasma choline, betaine, dimethylglycine (DMG), and trimethylamine *N*-oxide (TMAO) were measured using LC-MS/MS stable isotope dilution methods as described by Yan et al. [24].

### 2.6. Statistical Analysis

Differences between the two folic acid supplementation groups at each time point were assessed by unpaired *t*-tests (continuous dependent variables). Differences in values of maternal and cord blood pairs were assessed by paired *t*-tests using GraphPad Prism version 5 (La Jolla, CA, USA). Repeated measures analyses were conducted using SAS version 9.3 (Cary, NC, USA). Blood concentration response to folic acid supplementation was analyzed using a mixed effects model where correlations among repeated measures were considered to examine response to supplementation over time. Potential confounding variables included race/ethnicity, MTHFR genotype, pre-pregnancy BMI, gestational age at enrollment, and duration of previous folic acid supplementation use. Potential effects were modeled individually by repeated measures analysis of covariance using mixed effect models. The level of statistical significance was defined at *p* < 0.05. 

## 3. Results

### 3.1. Participant Characteristics

Fifty-one pregnant women were recruited at their initial prenatal visit. Out of the 51 pregnant women, 23 women discontinued the study during the intervention period. Reasons for discontinuing included morning sickness (n = 8), taking other prenatal supplements (n = 2), moving to another prenatal clinic (n = 2), non-singleton pregnancy (n = 1), failure to comply with study protocols (n = 1), and miscarriage (n = 1), and 8 women dropped for unknown reasons. Thus, 28 participants completed the intervention and were retained through delivery with 92.1% compliance. Among the participants who completed the study, 16 had been randomized to take 400 μg/day of folic acid and 12 had been randomized to take 800 μg/day of folic acid. Clinical and demographic characteristics of these participants are summarized in Table 1. There was no difference in clinical measures at baseline or delivery between groups; however, the 400 μg/day group had higher pre-pregnancy BMI based on self-report. Women in this group also had higher body weight at baseline, but there was no significant difference in BMI between groups at baseline.

Eight women from each group (16 total) reported taking a supplement containing folic acid prior to study baseline. In the 400 μg/day group, 25% of mothers and 15.4% of infants had the TT polymorphism for the MTHFR genotype, which indicates higher risk for folate deficiency. In the 800 μg/day group, 8.3% of mothers and 8.3% of infants had the TT polymorphism. Mothers with the TT genotype were unrelated to infants with the same genotype. There were no differences in average intake of total calories between the 400 and 800 μg/day groups (1971.9 ± 201.6 kcal vs. 2329.3 ± 322.4 kcal, respectively), food folate (220.4 ± 28.3 μg vs. 279.1 ± 29.0 μg), food folic acid (167.0 ± 19.6 μg vs. 245.9 ± 29.0 μg), dietary folate equivalents (504.3 ± 52.0 μg vs. 697.0 ± 102.1 μg), or average intake of vitamin B12 (5.2 ± 0.6 μg vs. 5.8 ± 1.6 μg), protein (81.5 ± 8.3 g vs. 95.0 ± 12.5 g), iron (14.7 ± 1.1 mg vs. 18.5 ± 3.2 mg), or fiber (19.3 ± 3.8 g vs. 22.1 ± 2.5 g) between groups during the intervention period (*p* > 0.05).

Infant outcomes are summarized in Table 2. There were no differences between groups except for the one-minute Apgar score, where the 400 μg/day group was significantly higher (*p* = 0.0411). This difference disappeared at five minutes and all Apgar scores at both time points fell within the normal range. 

### 3.2. Effect of Folic Acid Dose During Pregnancy on Folate Biomarkers in Maternal and Cord Blood

**Figure 2:**
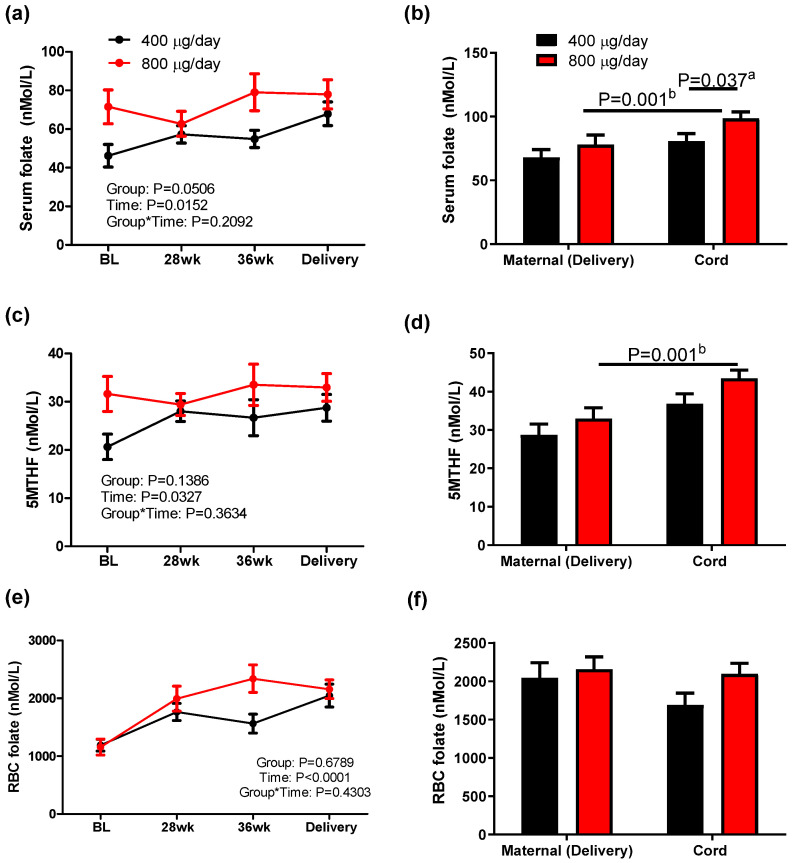
Serum folate (**a**,**b**), 5MTHF (5-ethyltetrahydrofolate, (**c**,**d**)) and RBC (red blood cell) folate (**e**,**f**) concentration in maternal and cord blood in response to 400 or 800 μg/day folic acid supplementation during pregnancy. Mean ± sem, ^a^ two-tailed unpaired *t*-test between groups of infants, ^b^ two-tailed paired *t*-test between mother–infant pairs.

Serum folate, plasma 5-Methyltetrahydrofolate (5MTHF) and RBC folate responses were measured to investigate the effect of folic acid dose during pregnancy on folate status. The responses of serum folate and plasma 5MTHF to supplemental folic acid dose were similar. A repeated measures analysis of variance of serum folate and plasma 5MTHF concentrations identified no significant group or group–time effects but did indicate a significant time effect. Women in the 800 μg/day group had higher concentrations of serum folate compared to the 400 μg/day group at baseline and 36 weeks, but this difference disappeared at delivery, as shown in Figure 2a,c. Mixed effects models were utilized to investigate potential confounding factors including the maternal MTHFR genotype, pre-pregnancy BMI, length of folic acid supplementation before enrolling in the study, and gestational age at baseline. Pre-pregnancy BMI had a significant overall effect on serum folate (*p* = 0.0360) and plasma 5MTHF (*p* = 0.0419). After adjusting for pre-pregnancy BMI, the group effect became significant (*p* = 0.0407 and *p* = 0.0497 for serum folate and plasma 5MTHF, respectively). Because serum folate reflects short-term folate status, previous folic acid supplementation may have affected concentrations. Participants who did not previously supplement with folic acid tended to have an increase in serum folate and plasma 5MTHF over time, while participants who reported previous supplementation did not demonstrate this increase during pregnancy, likely because of their higher baseline folate status. The cord blood in the 800 μg/day group also had significantly higher serum folate than cord blood in the 400 μg/day group (*p* = 0.037, Figure 2b). Cord blood in the 800 μg/day group had significantly higher concentrations of serum folate and plasma 5MTHF than mothers in the same group (Figure 2d), but there was no difference in maternal and cord concentrations in the 400 μg/day group.

Responses of RBC folate concentration to folic acid dose during pregnancy were measured to represent long-term folate status. RBC folate concentrations increased during gestation in both groups, but no group effect was observed by a repeated measures analysis of variance (Figure 2e). None of the variables considered for the mixed effect model analysis had an overall effect on RBC folate, and the time effect observed on RBC folate remained significant after adjusting for these variables, as shown in Figure 2e. Additionally, folic acid dose during pregnancy did not affect RBC folate concentration in maternal or cord blood (Figure 2f).

### 3.3. Effect of Folic Acid Dose During Pregnancy on Unmetabolized Folic Acid in Maternal and Cord Blood

**Figure 3:**
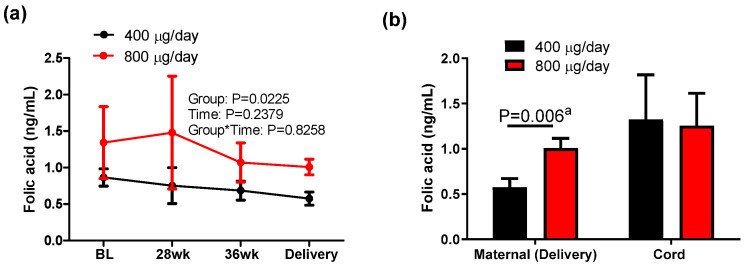
Unmetabolized folic acid concentration in maternal and cord blood in response to 400 or 800 μg/day folic acid supplementation during pregnancy. Mean ± sem, ^a^ two-tailed unpaired *t*-test between groups of mothers.

Serum unmetabolized folic acid in maternal and cord blood was measured in response to folic acid dose during pregnancy. Detectable amounts of folic acid were present in all maternal and cord blood samples, with a limit of detection (LOD) of 0.075 ng/mL. There was a significant group effect (*p* = 0.0225) on serum folic acid (Figure 3a), indicating that women supplemented with 800 μg/day folic acid during pregnancy maintained higher serum folic acid concentrations compared to those supplemented with 400 μg/day folic acid. Accordingly, at delivery, the 800 μg/day group had significantly higher concentrations than the 400 μg/day group (*p* = 0.006, Figure 3b). However, there was no difference in serum folic acid in cord blood between two groups. These findings suggest that folic acid dose impacts the concentration of unmetabolized folic acid in mothers, but not infants, and that a mechanism may exist to maintain a steady fetal concentration.

### 3.4. Effect of Folic Acid Dose During Pregnancy on Choline and Choline Metabolites in Maternal and Cord Blood

**Figure 4:**
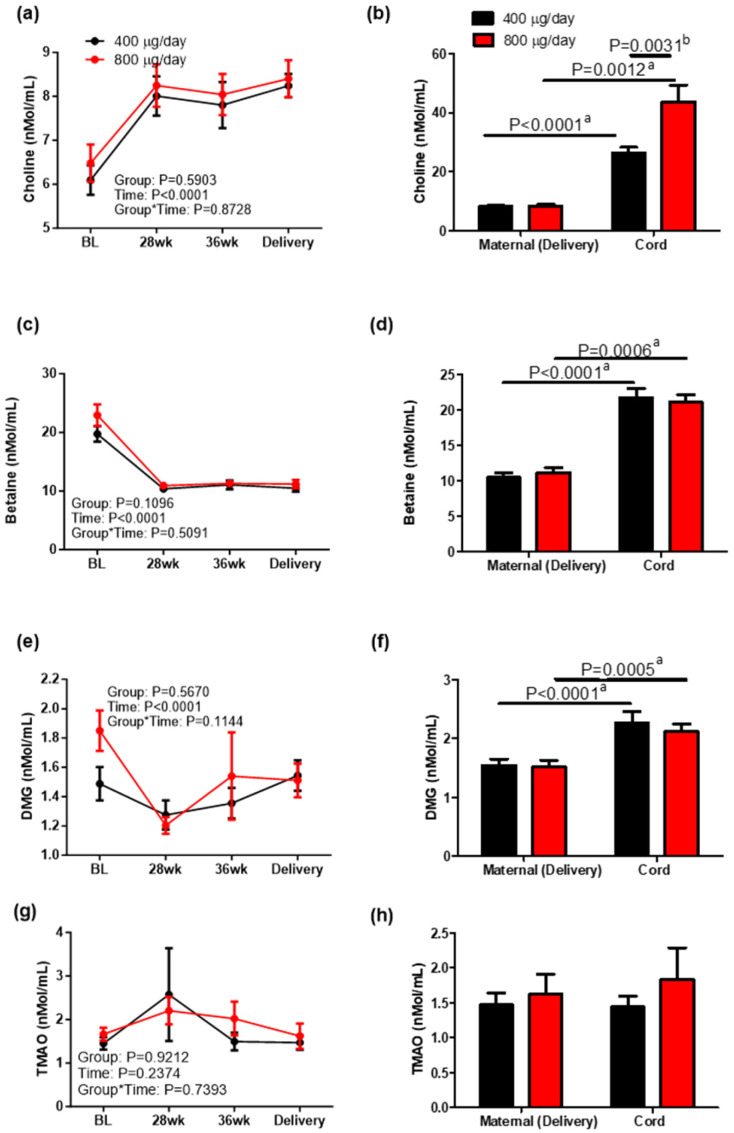
Choline (**a**,**b**), betaine (**c**,**d**), DMG (dimethylglycine, (**e**,**f**)) and TMAO (**g**,**h**) concentration in maternal and cord blood in response to 400 or 800 μg/day folic acid supplementation during pregnancy. Mean ± sem, ^a^ two-tailed unpaired *t*-test between groups of mothers or groups of infants, ^b^ two-tailed paired *t*-test between mother–infant pairs.

To explore the effect of folic acid dose on one-carbon metabolism in addition to folate status, plasma choline and its metabolites betaine, DMG and TMAO were measured as shown in Figure 4. There was no group or group–time effect on plasma choline (Figure 4a) or choline metabolites (Figure 4c,e,g). A time effect was observed on plasma choline, betaine and DMG. Plasma choline increased at 28 weeks and remained higher than baseline until delivery in both groups, while betaine was decreased at 28 weeks compared to that at baseline and maintained the level until delivery. Plasma DMG decreased at 28 weeks and showed a U-shaped response across gestation in both groups. Cord blood plasma choline concentrations were higher in the 800 μg/day group than in the 400 μg/day group (*p* = 0.0031), suggesting that maternal folic acid dose during pregnancy may affect cord blood choline metabolism while having no effect on maternal plasma choline at delivery. Cord blood concentrations of choline, betaine, and DMG were significantly higher than blood concentrations in their mothers (*p* < 0.05), consistent with previous studies [24,25].

## 4. Discussion

The current study was performed to determine changes in unmetabolized folic acid concentrations and one-carbon biomarkers in pregnant women and their infants in response to folic acid supplementation with either 400 μg/day (equivalent to the current RDA) or 800 μg/day (the amount commonly found in over-the-counter prenatal supplements) throughout pregnancy. To our knowledge, this is the first randomized controlled intervention trial comparing folate biomarkers in response to these two levels of folic acid supplementation from ~12 weeks of gestation to delivery and in cord blood. The results indicated that pregnant women who took 800 μg/day folic acid had higher concentrations of unmetabolized folic acid than women in the 400 μg/day group at delivery. Conversely, there was no difference in unmetabolized folic acid concentrations in cord blood between the two groups, suggesting the presence of a placental mechanism maintaining fetal concentrations. In addition, choline concentrations were higher in cord blood from the 800 μg/day group, suggesting that higher folate availability could have a sparing effect on cord blood choline.

In the current study, unmetabolized folic acid was detectable in all maternal samples and cord blood samples. Previous studies examining unmetabolized folic acid concentrations in pregnant women and cord blood reported a lower prevalence of detectable unmetabolized folic acid in the maternal and cord blood samples [10,19]. Obeid et al. detected unmetabolized folic acid in 43.6% of their pregnant participants and 54.2% of cord blood samples [10]. In the study by Pentieva et al. [19], 33–42% of maternal blood samples and only 20% of cord blood samples contained a detectable amount of unmetabolized folic acid. The difference in results between the present study and the studies by Obeid et al. and Pentieva et al. may be due the greater sensitivity for detecting unmetabolized folic acid and differences in folic acid intake. In the current study, the LOD was 0.075 ng/mL (equivalent to 0.17 nmol/L) compared to 0.20 nmol/L and 0.27 nmol/L for the other two studies, respectively. Differences in the LOD were likely due to differences in values of signal-to-noise ratio in the studies, where the current study defined the LOD for a signal-to-noise ratio of 3 and the previously mentioned studies defined the LOD as a signal-to-noise ratio ≥ 5. All three studies used the same method of liquid chromatography–tandem mass spectrometry (LC-MS/MS) for analysis of unmetabolized folic acid in samples. Additionally, the two previous studies were performed in European countries where folic acid fortification is voluntary. Obeid et al. [10] did not report food folate or folic acid intake, and Pentieva et al. [19] reported lower intakes of food folate (182–186 μg/day vs. 220–279 μg/day, respectively), food folic acid (102–112 μg/day vs. 167–246 μg/day), and dietary folate equivalents (356–376 μg/day vs. 504–697 μg/day) compared to the current study. In contrast, a prospective study conducted in Canada [26], a country that mandates folic acid fortification, found similar results to the current study, reporting detectable unmetabolized folic acid in more than 90% of maternal and cord blood samples in pregnant women taking a supplement containing 400 μg/day folic acid. The slight difference between detectable unmetabolized folic acid in this study could be accounted for by their slightly less sensitive LOD being 0.2 nmol/L. Although the Canadian study was not a randomized control intervention, the results support the current study’s findings that supplementation and mandated fortification produce detectable unmetabolized folic acid in maternal and cord blood [26].

The impact of prenatal folic acid supplementation above the recommended dose on unmetabolized folic acid status is not fully understood. In our study, we compared daily supplementation with 400 μg or 800 μg folic acid and observed that 800 μg of folic acid resulted in higher maternal unmetabolized folic acid concentration throughout pregnancy. Due to the lack of a placebo group in our study, the result cannot be compared to the previous findings. However, it is possible that 800 μg of folic acid may exceed the capacity of dihydrofolate reductase (DHFR) to reduce dietary folic acid, resulting in an increase in the concentration of unmetabolized folic acid observed in our study. Although the biological relevance of unmetabolized folic acid in blood is unclear, concerns have been raised about the potential for high concentrations to result in adverse outcomes such as cancer, tumor development, reduced immune function, and more recently, ASD [7,27,28]. More research is needed to investigate the potential negative long-term health outcomes related to high intakes of folic acid during pregnancy.

Unmetabolized folic acid concentrations in cord blood were not different between groups in our study despite the increase in maternal unmetabolized folic acid concentration observed in the group supplemented with 800 μg/day of folic acid compared to the group supplemented with 400 μg/day. Similarly, a previous study comparing 400 μg/day of folic acid supplementation from 14 weeks of gestation to delivery to a placebo also found that unmetabolized folic acid status in cord blood was similar in the two groups [10,19]. Additionally, Plumptre et al. found a greater proportion of unmetabolized folic acid in total serum folate in maternal blood compared to cord blood (*p* < 0.0001), but no difference between unmetabolized folic acid concentrations in cord blood from mothers who took folic acid supplements compared to the cord blood of mothers who did not supplement [26].

Few studies have reported on the transport of unmetabolized folic acid [29], but human and animal studies suggest that even in the presence of high maternal intakes of certain nutrients, a mechanism exists to keep fetal concentrations stable. It is known that membranes within the placenta contain folate transporters that are regulated by signals from the mother, fetus, and placenta [12], and expression of placental receptors for certain nutrients in humans is regulated by neonatal concentrations, not maternal concentrations [13]. Thus, it is possible that certain placental nutrient transporters are signaled to limit the transport of nutrients after fetal concentrations reach a certain level. In animals, embryos of dams who consumed very high amounts of vitamin D had the same or lower concentrations as embryos of dams who took an amount that was adequate but ten times lower than the high dose [14]. It is possible that a similar mechanism may act to regulate placental receptors for unmetabolized folic acid and thereby maintain stable amounts of unmetabolized folic acid in the fetus.

Serum folate concentration reflects short-term folate status and is highly affected by recent folate intake and supplementation. Therefore, it is ideal to measure serum folate from fasting samples [30]. In our study, blood draws were conducted at the participants’ prenatal visits for which fasting was not required. Thus, the higher serum folate concentration we observed in the 800 μg/day folic acid group at baseline may have been due to previous folic acid supplementation or dietary intake prior to the blood draw. Data collected in our health behavior questionnaire administered shortly after enrolling in the study indicate that half of our participants (50%) supplemented with folic acid prior to enrolling in the study. Regardless of fasting status at the time of sampling, folic acid doses during pregnancy did not affect serum folate or 5MTHF concentration in mothers during pregnancy and at delivery, while a higher dose of folic acid supplementation increased cord blood folate concentrations.

Although there was no group effect on maternal serum folate response to folic acid dose during pregnancy, pre-pregnancy BMI had an overall effect on this biomarker. After adjusting for pre-pregnancy BMI, the group effect became significant for serum folate response. This suggests that pre-pregnancy BMI plays an important role in maternal serum folate status during pregnancy. Consistent with our findings, Shin et al. reported that women with lower pre-pregnancy BMI had higher serum folate concentrations than their counterparts with a BMI ≥ 30 [31]. Similarly, Han et al. reported lower serum folate concentrations in pregnant women who fell into the obese category pre-pregnancy than those who did not [32]. The negative correlation between serum folate concentration and BMI is also consistent with correlations observed in non-pregnant women of childbearing age, wherein NHANES data from 1988 to 1994 and 1999 to 2000 indicated that increased BMI in this population was associated with lower serum folate levels [33]. Although the mechanism of this disparity is not yet fully understood, it may be at least in part due to the hemodilution effect in people who are obese or overweight [34,35]. In addition, the findings of Martino et al. suggest that the association could potentially be related to reduced expression of FOLR1 receptors in obese mothers [36]. These associations indicate the potential need for overweight and obese women to take higher doses of folic acid to achieve serum folate levels equal to normal-weight women.

Choline is a vitamin that, along with folate, is involved in one-carbon metabolism and functions as a methyl donor. Although there were no differences in maternal choline concentrations during gestation in our study, cord blood samples in the 800 μg/day group had higher concentrations of choline than those in the 400 μg/day group. Visentin et al. [25] found that maternal free choline concentration was not a significant predictor of fetal free choline, a finding that is supported by the current study’s results. Based on this information, maternal folic acid intake and maternal folate status may play a role in signaling fetal uptake of choline. Some animal studies have also suggested that high folate availability in the body can spare choline because both function as methyl donors [37]. A study conducted in mice found that folate deficiency led to a decrease in choline concentrations but a dramatic increase in the reductase enzyme that converts choline to betaine, the compound that donates methyl groups [38]. This likely occurred to compensate for the low amounts of folate available. Consistent with these findings, choline concentrations in our study increased throughout pregnancy. The decrease in betaine was likely due to sufficient folate status as indicated by the increases in serum folate and 5MTHF concentrations, reducing the need for compensatory action. Higher folic acid supplementation during pregnancy may spare choline from use as a one-carbon donor and allow it to carry out other functions in the body including formation of acetylcholine, a neurotransmitter necessary for proper brain development [39].

Maternal nutrition during gestation plays a critical role in offspring metabolism, a phenomenon called fetal programming [40]. Because of folate’s involvement in DNA methylation, maternal folic acid/folate intake could affect infant health outcomes later in life including cardiovascular health, fat accumulation, insulin resistance, and glycemic control. In our study, there were no differences in the clinical characteristics of infants between the two groups except for one-minute Apgar score, which is not clinically relevant because all infants in both groups had Apgar scores within the normal range both at one and five minutes. Because the current study only followed up until birth, there is potential for differences in long-term health outcomes between the two groups of infants, even if infant characteristics were not different at birth. Keating et al. found that Sprague Dawley rats fed a high folic acid diet had female offspring with higher weight gain, food intake, and impaired glycemic control compared to offspring of mothers fed a diet with adequate folic acid [41]. In humans, offspring of women who supplemented with folic acid had decreased DNA methylation associated with deregulation of IGF2 and increased risk of chronic disease compared to offspring of women who did not supplement [15]. These studies indicate the need for more long-term studies to investigate potential health consequences of maternal folic acid intake not evident at birth.

This study has several strengths. First, it was a randomized controlled intervention trial, ensuring unbiased results. It is also the only study to our knowledge that investigated outcomes in response to two specific folic acid doses throughout pregnancy: the RDA and the dose commonly found in prenatal supplements. It is also the first study that we are aware of comparing two doses of folic acid supplementation and unmetabolized folic acid concentrations in maternal and cord blood in a country with mandatory folic acid fortification. Folate and choline metabolism biomarkers were measured to better understand the relationship between these two compounds.

The current study is not without limitations. As mentioned previously, both women who did and did not supplement with folic acid prior to study baseline were included, which may have influenced our results. Ideally, a follow up study would control for previous supplementation, considering the fact that periconceptual folic acid supplementation is critically important since the neural tubes in the embryo close about four weeks after conception and current recommendations emphasize pre- and peri-conceptual folic acid supplementation. Blood samples were also non-fasting. Additionally, the explanations for the results presented are speculative because physiological mechanisms were not investigated. Finally, because this trial was only conducted through childbirth, long-term effects in mothers or infants related to supplementation with 400 or 800 μg/day folic acid are unknown.

## 5. Conclusions

In summary, supplementation with 800 μg/day folic acid throughout pregnancy did not affect serum folate or RBC folate concentrations but did increase unmetabolized folic acid concentrations in mothers compared to the 400 μg/day dose. Conversely, the higher dose of folic acid supplementation during pregnancy increased serum folate but not serum unmetabolized folic acid concentrations in cord blood. Although there was no group difference with choline concentrations in maternal samples, cord blood samples from the 800 μg/day group had higher choline concentrations. Taken together, folic acid dose during pregnancy had a distinct influence on folate biomarkers in maternal and cord blood. Potential mechanisms for these disparities may involve the regulatory roles of the placenta, pointing to the need for studies designed to investigate placental regulation of one-carbon biomarkers between maternal and cord blood. Further studies are warranted to investigate long-term effects of increased unmetabolized folic acid in mothers as well as the effects of increased serum folate and choline in infants in response to folic acid doses contained in over-the-counter prenatal vitamins.

## Figures and Tables

**Table 1:** Demographic and clinical characteristics of mothers ^a^.

		Baseline		Delivery	
		400 μg/Day (n = 16)	800 μg/Day (n = 12)	400 μg/Day (n = 16)	800 μg/Day (n = 12)
Age (years)		28.3 ± 1.5	26.6 ± 1.2	-	-
Weight (kg)		75.1 ± 3.6	62.9 ± 3.1 *	86.3 ± 3.0 ^+^	76.5 ± 2.3 *^+^
Height (cm)		166.0 ± 2.0	161.8 ± 1.9	-	-
BMI (kg/m^2^)		27.2 ± 1.2	24.1 ± 1.3	31.3 ± 0.9 ^+^	29.1 ± 0.9 ^+^
Gestational Age (weeks)		7.7 ± 0.3	7.1 ± 0.6	40.1 ± 0.2 ^+^	40.0 ± 0.4 ^+^
Pre-Pregnancy BMI (kg/m^2^)		27.0 ± 1.2	23.3 ± 1.3 *	-	-
Hemoglobin (g/dL)		13.0 ± 0.2	12.9 ± 0.8	12.0 ± 0.4	12.5 ± 1.0
Hematocrit (%)		39.2 ± 0.7	39.4 ± 3.0	36.6 ± 1.2 ^+^	36.8 ± 1.95 ^+^
RBC (1 × 10^6^/μL)		4.5 ± 0.1	4.3 ± 0.4	4.1 ± 0.1	4.1 ± 0.4
WBC (1 × 10^3^/μL)		8.5 ± 2.6	8.0 ± 2.7	10.2 ± 3.0 ^+^	12.2 ± 3.4 ^+^
Previous FA Supplementation	Yes	n = 8 (50%)	n = 8 (66%)	-	-
	No	n = 5 (31%)	n = 2 (17%)	-	-
	Unknown	n = 3 (19%)	n = 2 (17%)	-	-
Race/Ethnicity	White	n = 7 (44%)	n = 7 (58%)	-	-
	Hispanic	n = 5 (31%)	n = 4 (33%)	-	-
	African–American	n = 3 (18%)	n = 0 (0%)	-	-
	Other	n = 1 (7%)	n = 1 (9%)	-	-
MTHFR Genotype	CC	n = 9 (56%)	n = 8 (66%)	-	-
	TC	n = 3 (19%)	n = 3 (25%)	-	-
	TT	n = 4 (25%)	n = 1 (9%)	-	-

^a^ Mean ± standard deviation, * Groups are significantly different (*p* < 0.05) at baseline or delivery by paired *t*-test, ^+^ baseline and delivery values are significantly different (*p* < 0.05) using unpaired *t*-test.

**Table 2:** Clinical characteristics of infants ^a^.

		400 μg/Day (n = 16)	800 μg/Day (n = 12)
Length (cm)		51.3 ± 0.5	51.7 ± 0.5
Weight (kg)		3.6 ± 0.1	3.5 ± 0.1
Head Circumference (cm)		33.6 ± 1.4	33.9 ± 0.5
Gestational Age at delivery		40.1 ± 0.2	40.0 ± 0.4
Apgar—1 min		8.3 ± 0.2	7.6 ± 0.3 *
Apgar—5 min		8.8 ± 0.1	8.5 ± 0.4
Hemoglobin (g/dL)		16.2 ± 1.8	14.5 ± 1.5
Hematocrit (%)		48.4 ± 5.6	48.0 ± 7.4
RBC (1 × 10^6^/μL)		4.6 ± 0.6	4.3 ± 0.2
WBC (1 × 10^3^/μL)		12.6 ± 4.8	14.3 ± 4.6
MTHFR Genotype	CC	n = 4 (25%)	n = 7 (58%)
	TC	n = 7 (44%)	n = 4 (33%)
	TT	n = 2 (13%)	n = 1 (9%)

^a^ Mean ± standard deviation, * Groups are significantly different (*p* < 0.05) by unpaired *t*-test.

## Data Availability

The data presented in this study are available on request from the corresponding author due to privacy or ethical restrictions.

## References

[1] Zhao R., Matherly L.H., Goldman I.D. (2009). Membrane transporters and folate homeostasis: Intestinal absorption and transport into systemic compartments and tissues. Expert Rev. Mol. Med..

[2] Stover P.J., Bailey L. (2010). Folate biochemical pathways and their regulation. Folate in Health and Disease.

[3] Tamura T., Picciano M.F. (2006). Folate and human reproduction. Am. J. Clin. Nutr..

[4] Cunningham F.G., MacDonald P.C., Gant N.F., Leveno K.G., Gilstrap L.C. (1989). Physiology in pregnancy. Williams Obstetrics.

[5] Institute of Medicine (1998). The National Academies Collection: Reports funded by National Institutes of Health. Dietary Reference Intakes for Thiamin, Riboflavin, Niacin, Vitamin B6, Folate, Vitamin B12, Pantothenic Acid, Biotin, and Choline.

[6] Kauwell G.P., Diaz M.L., Yang Q., Bailey L.B. (2010). Recommended intakes, consumption, and status. Folate in Health and Disease.

[7] Smith A.D., Kim Y.I., Refsum H. (2008). Is folic acid good for everyone?. Am. J. Clin. Nutr..

[8] Kelly P., McPartlin J., Goggins M., Weir D.G., Scott J.M. (1997). Unmetabolized folic acid in serum: Acute studies in subjects consuming fortified food and supplements. Am. J. Clin. Nutr..

[9] Pfeiffer C.M., Sternberg M.R., Fazili Z., Yetley E.A., Lacher D.A., Bailey R.L., Johnson C.L. (2015). Unmetabolized folic acid is detected in nearly all serum samples from US children, adolescents, and adults. J. Nutr..

[10] Obeid R., Kasoha M., Kirsch S.H., Munz W., Herrmann W. (2010). Concentrations of unmetabolized folic acid and primary folate forms in pregnant women at delivery and in umbilical cord blood. Am. J. Clin. Nutr..

[11] Sweeney M.R., McPartlin J., Weir D.G., Daly S., Pentieva K., Daly L., Scott J.M. (2005). Evidence of unmetabolised folic acid in cord blood of newborn and serum of 4-day-old infants. Br. J. Nutr..

[12] Lager S., Powell T.L. (2012). Regulation of nutrient transport across the placenta. J. Pregnancy.

[13] Young B.E., Cooper E.M., McIntyre A.W., Kent T., Witter F., Harris Z.L., O’Brien K.O. (2014). Placental vitamin D receptor (VDR) expression is related to neonatal vitamin D status, placental calcium transfer, and fetal bone length in pregnant adolescents. FASEB J..

[14] Costabile B.K., Kim Y.K., Iqbal J., Zuccaro M.V., Wassef L., Narayanasamy S., Curley R.W., Harrison E.H., Hussain M.M., Quadro L. (2016). Beta-Apo-10′-carotenoids modulate placental microsomal triglyceride transfer protein expression and function to optimize transport of intact beta-Carotene to the embryo. J. Biol. Chem..

[15] Hoyo C., Murtha A.P., Schildkraut J.M., Jirtle R.L., Demark-Wahnefried W., Forman M.R., Iversen E.S., Kurtzberg J., Overcash F., Huang Z. (2011). Methylation variation at IGF2 differentially methylated regions and maternal folic acid use before and during pregnancy. Epigenetics.

[16] Xu X., Zhang Z., Lin Y., Xie H. (2024). Risk of Excess Maternal Folic Acid Supplementation in Offspring. Nutrients.

[17] Raghavan R., Riley A.W., Volk H., Caruso D., Hironaka L., Sices L., Hong X., Wang G., Ji Y., Brucato M. (2018). Maternal Multivitamin Intake, Plasma Folate and Vitamin B12 Levels and Autism Spectrum Disorder Risk in Offspring. Paediatr. Perinat. Epidemiol..

[18] Raghavan R., Selhub J., Paul L., Ji Y., Wang G., Hong X., Zuckerman B., Fallin M.D., Wang X. (2020). A prospective birth cohort study on cord blood folate subtypes and risk of autism spectrum disorder. Am. J. Clin. Nutr..

[19] Pentieva K., Selhub J., Paul L., Molloy A.M., McNulty B., Ward M., Marshall B., Dornan J., Reilly R., Parle-McDermott A. (2016). Evidence from a randomized trial that exposure to supplemental folic acid at recommended levels during pregnancy does not lead to increased unmetabolized folic acid concentrations in maternal or cord blood. J. Nutr..

[20] Subar A.F., Kirkpatrick S.I., Mittl B., Zimmerman T.P., Thompson F.E., Bingley C., Willis G., Islam N.G., Baranowski T., McNutt S. (2012). The Automated Self-Administered 24-hour dietary recall (ASA24): A resource for researchers, clinicians, and educators from the National Cancer Institute. J. Acad. Nutr. Diet..

[21] Shade D.C., Park H.J., Hausman D.B., Hohos N., Meagher R.B., Kauwell G.P.A., Kilaru V., Lewis R.D., Smith A.K., Bailey L.B. (2017). DNA Methylation Changes in Whole Blood and CD16+ Neutrophils in Response to Chronic Folic Acid Supplementation in Women of Childbearing Age. Int. J. Vitam. Nutr. Res..

[22] Horne D.W., Patterson D. (1988). Lactobacillus casei microbiological assay of folic acid derivatives in 96-well microtiter plates. Clin. Chem..

[23] West A.A., Yan J., Perry C.A., Jiang X., Malysheva O.V., Caudill M.A. (2012). Folate-status response to a controlled folate intake in nonpregnant, pregnant, and lactating women. Am. J. Clin. Nutr..

[24] Yan J., Jiang X., West A.A., Perry C.A., Malysheva O.V., Devapatla S., Pressman E., Vermeylen F., Stabler S.P., Allen R.H. (2012). Maternal choline intake modulates maternal and fetal biomarkers of choline metabolism in humans. Am. J. Clin. Nutr..

[25] Visentin C.E., Masih S., Plumptre L., Malysheva O., Nielsen D.E., Sohn K.J., Ly A., Lausman A.Y., Berger H., Croxford R. (2015). Maternal choline status, but not fetal genotype, influences cord plasma choline metabolite concentrations. J. Nutr..

[26] Plumptre L., Masih S.P., Ly A., Aufreiter S., Sohn K.-J., Croxford R., Lausman A.Y., Berger H., O’Connor D.L., Kim Y.-I. (2015). High concentrations of folate and unmetabolized folic acid in a cohort of pregnant Canadian women and umbilical cord blood. Am. J. Clin. Nutr..

[27] Stolzenberg-Solomon R.Z., Chang S.C., Leitzmann M.F., Johnson K.A., Johnson C., Buys S.S., Hoover R.N., Ziegler R.G. (2006). Folate intake, alcohol use, and postmenopausal breast cancer risk in the Prostate, Lung, Colorectal, and Ovarian Cancer Screening Trial. Am. J. Clin. Nutr..

[28] Troen A.M., Mitchell B., Sorensen B., Wener M.H., Johnston A., Wood B., Selhub J., McTiernan A., Yasui Y., Oral E. (2006). Unmetabolized folic acid in plasma is associated with reduced natural killer cell cytotoxicity among postmenopausal women. J. Nutr..

[29] Henderson G.I., Perez T., Schenker S., Mackins J., Antony A.C. (1995). Maternal-to-fetal transfer of 5-methyltetrahydrofolate by the perfused human placental cotyledon: Evidence for a concentrative role by placental folate receptors in fetal folate delivery. J. Lab. Clin. Med..

[30] Shane B. (2010). Folate Chemistry and Metabolism.

[31] Shin D., Lee K.W., Song W.O. (2016). Pre-pregnancy weight status is associated with diet quality and nutritional biomarkers during pregnancy. Nutrients.

[32] Han Y.S., Ha E.H., Park H.S., Kim Y.J., Lee S.S. (2011). Relationships between pregnancy outcomes, biochemical markers and pre-pregnancy body mass index. Int. J. Obes..

[33] Mojtabai R. (2004). Body mass index and serum folate in childbearing age women. Eur. J. Epidemiol..

[34] Cepeda-Lopez A.C., Zimmermann M.B., Wussler S., Melse-Boonstra A., Naef N., Mueller S.M., Toigo M., Herter-Aeberli I. (2019). Greater blood volume and Hb mass in obese women quantified by the carbon monoxide-rebreathing method affects interpretation of iron biomarkers and iron requirements. Int. J. Obes..

[35] da Silva V.R., Hausman D.B., Kauwell G.P., Sokolow A., Tackett R.L., Rathbun S.L., Bailey L.B. (2013). Obesity affects short-term folate pharmacokinetics in women of childbearing age. Int. J. Obes..

[36] Martino J., Segura M.T., García-Valdés L., Padilla M.C., Rueda R., McArdle H.J., Budge H., Symonds M.E., Campoy C. (2018). The impact of maternal pre-pregnancy body weight and gestational diabetes on markers of folate metabolism in the placenta. Nutrients.

[37] Schwahn B.C., Laryea M.D., Chen Z., Melnyk S., Pogribny I., Garrow T., James S.J., Rozen R. (2004). Betaine rescue of an animal model with methylenetetrahydrofolate reductase deficiency. Biochem. J..

[38] Chew T.W., Jiang X., Yan J., Wang W., Lusa A.L., Carrier B.J., West A.A., Malysheva O.V., Brenna J.T., Gregory J.F. (2011). Folate intake, MTHFR genotype, and sex modulate choline metabolism in mice. J. Nutr..

[39] Zeisel S.H., Klatt K.C., Caudill M.A. (2018). Choline. Adv. Nutr..

[40] Chmurzynska A. (2010). Fetal programming: Link between early nutrition, DNA methylation, and complex diseases. Nutr. Rev..

[41] Keating E., Correia-Branco A., Araujo J.R., Meireles M., Fernandes R., Guardao L., Guimaraes J.T., Martel F., Calhau C. (2015). Excess perigestational folic acid exposure induces metabolic dysfunction in post-natal life. J. Endocrinol..

